# Case Report: Identification of Germline Chimerism in Monochorionic Dizygotic Twins

**DOI:** 10.3389/fgene.2021.744890

**Published:** 2021-11-19

**Authors:** Juan Chen, Jian Xu, Zhi-Heng Chen, Min-Na Yin, Xin-Yu Guo, Ling Sun

**Affiliations:** ^1^ Department of Assisted Reproductive technology, Guangzhou Women and Children’s Medical Center, Guangzhou Medical University, Guangzhou, China; ^2^ Reproductive Medicine Center, Department of Obstetrics and Gynecology, Guangzhou General Hospital of Guangzhou Military Region, Guangzhou, China

**Keywords:** MCDZ twins, tissues chimerism, germ cell, PGT-A, ddPCR

## Abstract

Monochorionic twins are generally considered to be monozygotic, as monochorionic dizygotic (MCDZ) twins are extremely rare in natural pregnancies. Several studies have reported this rare occurrence, and most of these pregnancies have been conceived by assisted reproductive technology (ART). These reports mostly focused on MCDZ twin pregnancies and the childhood development of the twins; a follow-up into adulthood and the effect on their reproduction has not been reported. In this case study, we report a case of chimerism in opposite-sex MCDZ twins who were naturally conceived and have reached reproductive maturity. We collected oral mucosal, endometrial, and germ cells from the twins and evaluated their chimerism using single-nucleotide polymorphism (SNP) array and droplet digital PCR (ddPCR). The SNP array showed that they had 4,049 non-allele shared loci, and they inherited nearly 50% informative SNP loci from each parent, confirming that they are dizygotic twins. We found that the female twin had a 46, XX (2)/46, XY (78) karyotype in her peripheral blood. The SNP array confirmed that the female twin and male twin had the same blood haplotype. The ddPCR result showed 92.84 (± 1.80%) chimerism in her blood. In case of chimerism in her germline, the female twin chose preimplantation genetic testing for aneuploidy for her blastocysts. Fortunately, the patient only had blood chimerism. A healthy boy was born at 39 weeks of gestation.

## Introduction

Monochorionic twins are generally considered to be monozygotic. However, monochorionic dizygotic (MCDZ) twins, an extremely rare occurrence, have been reported due to rapidly improving cytogenetic and DNA sequencing techniques (Nylander and Osunkoya, 1970; Lee et al., 2014a; Rodriguez-Buritica et al., 2015; Korsun et al., 2016; Uysal et al., 2018; Gabbett et al., 2019). MCDZ twins share the same placenta, which leads to cell exchange between embryos with different genotypes *via* vascular anastomoses, resulting in blood chimerism. In theory, the cell exchange between two fetuses only occurs in blood cells. However, chimerism in other tissues has been reported in several studies (Fumoto et al., 2014; Rodriguez-Buritica et al., 2015; Korsun et al., 2016; Uysal et al., 2018), and the early-stage fusion of dizygotic embryos was speculated to be the reason for occurrence. The presence of tissue chimerism might lead to serious clinical consequences in MCDZ twins, especially in opposite-sex MCDZ twins. When chimerism occurs, the karyotype and genotype in the blood cannot present other tissue status. In 2003, Johannsen et al. (2003) reported a case of erroneous sex classification that led to ovary removal in a twin girl since she presented with a 46, XY blood karyotype and was suspected of having gonadal dysgenesis. In addition, when blood chimerism occurs, it might lead to an incorrect interpretation of blood group phenotypes in blood donors (Bluth et al., 2007), increasing the risk of a hemolytic reaction in recipients (Parva et al., 2009; Assaf et al., 2010). Most reports focus on MCDZ twin pregnancies, and they follow up on their childhood development for a few years. A report on adult MCDZ twins and the effect on their reproduction is rare, as most of the reported cases were recently conceived by assisted reproductive technology (ART). Here, we present naturally conceived, opposite-sex MCDZ twins who reached reproductive maturity; we investigated their chimerism in different tissues and germline cells. This information will assist researchers and clinicians in evaluating the clinical consequences of chimerism and raising awareness on the condition.

## Case Description

A 27-year-old woman (twin 2) who miscarried once in 2018 visited our center to consult for infertility. Her physical examination showed normal breast and internal and external genitalia development. She had menarche at 14 years of age, and her menstruation cycles were regular. Basal hormone levels and anti-Müllerian hormone (AMH; 5.05 ng/ml) levels were normal. A karyotype analysis of her peripheral blood showed a 46, XX (2)/46, XY (78) chimeric karyotype. She claimed that she has never received a blood transfusion before, but she does have a twin brother (twin 1). Her mother informed us that she had one large placenta when she gave birth to the twins. We assumed that the patient’s chimerism came from her twin brother and we informed her of her blood chimerism and germline chimerism risk. After informed consent was obtained, we collected buccal cells and blood from the twins. Additionally, sperm cells were collected from her twin brother. DNA was extracted and a single-nucleotide polymorphism (SNP) array (Karyomapping, Illumina) was performed.

**FIGURE 1:**
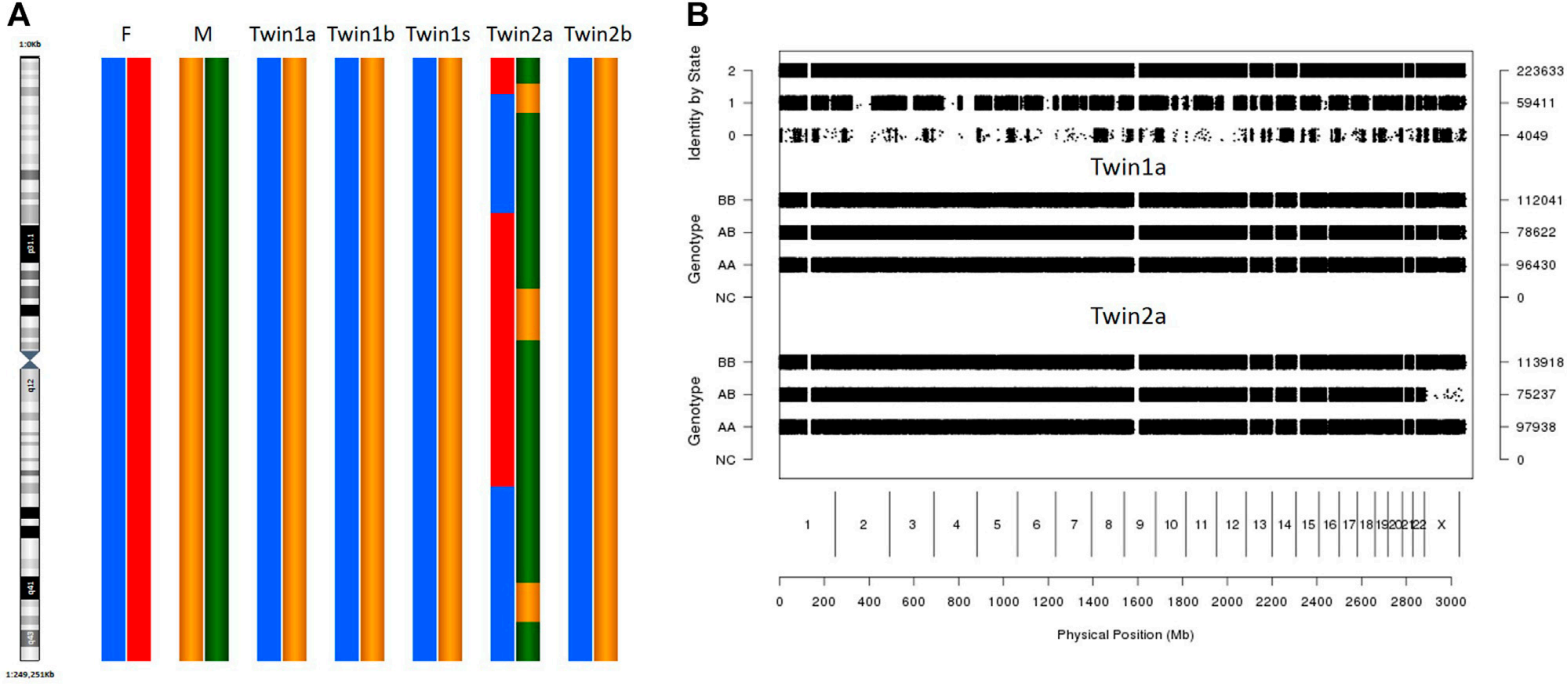
SNP array result of different tissues. **(A)** Karyomapping (chr1) of different tissues in twins. F: father M: mother. Twin1a/b/s: Oral mucosa cells, blood, and sperm in twin1. Twin2 a/b: Oral mucosa cells, blood in twin2. **(B)** Result of pairwise SNP analysis in genome wide. SNP analysis used the SNPduo Web tool. Identity by state (IBS) 2: both alleles shared between twins; IBS1: single allele shared between twins; IBS0: non-allele shared. Twin1a: Oral mucosa cells twin1. Twin2 a: Oral mucosa cells in twin2.

The karyomapping results showed that twin 2 had different genetic karyotypes between her oral mucosal cells and blood cells (Figure 1A), and her blood karyotype was identical to that of twin 1. The karyomapping results also indicated which alleles are shared by the twins (Figure 1B). They have 4,049 non-allele-shared loci, which means that they were conceived by different oocytes and sperms. They inherited nearly 50% informative SNP loci from each parent (data not shown), confirming that they are dizygotic twins.

Based on the karyomapping results, we designed 23 pairs of ddPCR primers (Supplementary Table S1) to distinguish chimerism in different tissues (oral mucosa, endometrium, and germline cells). The results indicated that twin 1 has the same DNA in different tissues, and twin 2 has two sets of DNA in her blood with 92.84 ± 1.80% chimerism (Table 1). There was no chimerism detected in the oral mucosa or endometrium of twin 2 (Table 1).

**TABLE 1:** ddPCR result of different tissue in twin 1 and twin 2.

Tissue		Twin 1 (%)	Twin 2 (%)
Twin1	Blood	100.29 ± 1.57	-0.29 ± 1.57
	Oral mucosa	99.3 ± 13.33	0.7 ± 13.33
	Sperm	100.76 ± 2.51	-0.76 ± 2.51
Twin2	Blood	92.84 ± 1.80	7.16 ± 1.80
	Oral mucosa	0.49 ± 1.83	99.51 ± 1.83
	Endometrium	−0.09 ± 1.16	100.09 ± 1.16
	Oocyte 1^a^	1.18 ± 3.18	98.82 ±3.18
	Oocyte 2^a^	0.77 ± 2.19	99.23 ± 2.19

All data are presented as the mean ± SD.

a Only 11 sets of Twin 2 informative SNP probes were used for oocytes.

In order to determine chimerism in germline cells, the patient chose preimplantation genetic testing for aneuploidy on her blastocysts. Thirty oocytes were retrieved from the ovaries of the patient, followed by fertilization *via* an intracytoplasmic sperm injection (ICSI). Twenty-one oocytes were successfully fertilized, and the zygotes were cultured in sequential G1/G2 media for 5–6 days. Ten blastocysts were obtained, and a biopsy of 5–10 cells from trophectoderm (TE) tissue was performed on day 5 or day 6, depending on the blastocyst stage. To confirm germline chimerism, two oocytes from failed fertilization and endometrial cells were collected. To obtain sufficient DNA for subsequent studies, whole genome amplification was performed on the TE biopsy and oocytes. Thereafter, the amplified DNA was dispensed onto Human CytoSNP-12 DNA BeadChips (Illumina, San Diego, CA, United States). Ten blastocysts and two oocytes were assessed for informative SNPs that were present in twin 1 but not in twin 2. Approximately, 0–5 out of the 300,000 SNPs were found in each blastocyst (data not shown). This result did not support the existence of chimerism in blastocysts and oocytes.

The SNP array was also analyzed for preimplantation genetic testing for aneuploidy testing. The results showed that seven embryos were euploid, one was aneuploid, and two were mosaic. A single euploid blastocyst was selected and transferred. At 12 weeks of gestation, non-invasive prenatal testing was performed and no aneuploidy was detected. A healthy boy was born at 39 weeks of gestation.

## Discussion

Several studies have reported chimerism in tissues other than the blood (Fumoto et al., 2014; Rodriguez-Buritica et al., 2015; Korsun et al., 2016; Uysal et al., 2018). However, in this case, chimerism was only observed in blood cells and not in other tissues. We assessed cervical epithelial cells from twin 2, and the initial ddPCR result showed the same karyotype as identified in her twin brother, indicating potential tissue chimerism. However, in the repeated experiment, we used flow cytometry to exclude leukocytes by staining with the anti-CD45 antibody, and it was found that most cells were CD45^+^. This means that most of the cervical epithelium cells we initially collected were leukocytes. Unfortunately, after cell sorting, the remainder CD45^−^ cells were not sufficient for further study.

In previous studies, there are two hypotheses to explain the mechanism of tissue chimerism, other than blood. One theory is that the fusion of dizygotic embryos at an early stage causes tissue chimerism. However, this theory is not supported by the developmental process of embryos. According to the embryology theory, the zygote is enclosed within the zona pellucida for at least 5 days until it develops into a blastocyst and hatches from the zona pellucida. Thus, the dizygotic embryo could not fuse until day five of embryogenesis. The blastocyst then divides into inner cell mass (fetal development) and trophectoderm (placental development). It is therefore unlikely that the inner mass of the blastocyst fuses and divides into two embryos. Another hypothesis is that tissue chimerism originates from bone marrow stem cells. The observation of bone marrow transplant patients led to this hypothesis. A study reported that after a female patient received a bone marrow transplant from a male donor (Taylor, 2004; Liu et al., 2018), the Y chromosome was detected in her endometrial cells. As a result, the author speculated that bone marrow stem cells are involved in endometrial regeneration. However, in our case this theory could not explain why, after numerous menstruation cycles, the endometrium of twin 2 never presented chimerism. As blood–cell contamination can occur in most tissue collections, the conclusion of tissue chimerism in MCDZ twins should be drawn cautiously.

An increasing number of MCDZ twins has been reported, and at least 80% of the reported cases were conceived by ART (Williams et al., 2004; Aoki et al., 2006; Ekelund et al., 2008; Parva et al., 2009; Assaf et al., 2010; Choi et al., 2013; Smeets et al., 2013; Lee et al., 2014a; Lee et al., 2014b; Fumoto et al., 2014; Korsun et al., 2016; Mayeur Le Bras et al., 2016). This indicates that when monochorionic twins, which originated from the transfer of two embryos, are diagnosed by ultrasound, we should consider two possibilities: the first possibility is that one embryo died and the other embryo developed to be monochorionic monozygotic (MCMZ), or alternatively, both embryos survive, share the placenta, and develop into MCDZ. Although MCDZ twinning is rare, this occurrence is expected to increase with the development of ART. Since same-sex MCDZ twinning is easily overlooked, the MCDZ incidence rate could be higher than previously reported. In order to avoid iatrogenic complications caused by chimerism, monochorionic twins originating from the transfer of two embryos should be recommended for the SNP array or other chromosomal screenings to exclude the possibility of blood chimerism.

In this study, we have gained some insight into MCDZ twins; however, there were limitations within this study. We only examined three different kinds of tissues and blood, and therefore, we could not rule out the possibility of chimerism in other tissues. Besides performing non-invasive prenatal testing, we did not test for tissue chimerism in the offspring of the patient. We will follow up on his condition in a subsequent study.

## Data Availability

The original contributions presented in the study are included in the article/Supplementary Material; further inquiries can be directed to the corresponding authors.
