# Supplementary material for: Case Report: Identification of Germline Chimerism in Monochorionic Dizygotic Twins
Source: Front Genet. 2021 Nov 19;12:744890. doi: 10.3389/fgene.2021.744890 (PMC8641794; doi:10.3389/fgene.2021.744890)
Supplement: Supplementary file 1 [file DataSheet2.docx]

**Suppl Table 1. SNPs positions and sequences**

| **Chr** | **Pos** | **SNP** | **FatherSeq** | **MotherSeq** | **Twin1** | **Twin2** | **Informative** | **region** |
| --- | --- | --- | --- | --- | --- | --- | --- | --- |
| **1** | **3446813** | **rs4648392** | **AG** | **GG** | **GG** | **AG** | **IFF** | **chr1:3446613-3447013** |
| **2** | **1384725** | **rs11897977** | **AA** | **AG** | **AG** | **AA** | **IFM** | **chr2:1384525-1384925** |
| **3** | **3109444** | **rs340831** | **TC** | **CC** | **CC** | **TC** | **IFF** | **chr3:3109244-3109644** |
| **4** | **16862164** | **rs1468546** | **TT** | **TC** | **TC** | **TT** | **IFM** | **chr4:16861964-16862364** |
| **5** | **3693627** | **rs11960785** | **TC** | **CC** | **CC** | **TT** | **IFF** | **chr5:3693427-3693827** |
| **6** | **37415125** | **rs9380673** | **TC** | **TT** | **TC** | **TT** | **IFF** | **chr6:37414925-37415325** |
| **7** | **2724098** | **rs10264843** | **CC** | **TC** | **CC** | **TC** | **IFM** | **chr7:2723898-2724298** |
| **8** | **22394920** | **rs1116085** | **AG** | **GG** | **AG** | **GG** | **IFF** | **chr8:22394720-22395120** |
| **9** | **3322283** | **rs3012708** | **AG** | **AA** | **AA** | **AG** | **IFF** | **chr9:3322083-3322483** |
| **10** | **10412208** | **rs10795792** | **TC** | **CC** | **TC** | **CC** | **IFF** | **chr10:10412008-10412408** |
| **11** | **36766061** | **rs11033786** | **GG** | **AG** | **AG** | **GG** | **IFM** | **chr11:36765861-36766261** |
| **12** | **10525644** | **rs1351113** | **GG** | **AG** | **GG** | **AG** | **IFM** | **chr12:10525444-10525844** |
| **13** | **23364220** | **rs2114224** | **AG** | **GG** | **AG** | **GG** | **IFF** | **chr13:23364020-23364420** |
| **14** | **38568732** | **rs12147319** | **AC** | **AA** | **AA** | **AC** | **IFF** | **chr14:38568532-38568932** |
| **15** | **32443813** | **rs7178176** | **TC** | **CC** | **TC** | **CC** | **IFF** | **chr15:32443613-32444013** |
| **16** | **50338723** | **rs9924714** | **TC** | **TT** | **TT** | **TC** | **IFF** | **chr16:50338523-50338923** |
| **17** | **6092546** | **rs8074059** | **AA** | **AC** | **AC** | **AA** | **IFM** | **chr17:6092346-6092746** |
| **18** | **5766006** | **rs12958345** | **AA** | **AG** | **AA** | **AG** | **IFM** | **chr18:5765806-5766206** |
| **19** | **17026448** | **rs11086039** | **TT** | **TC** | **TC** | **TT** | **IFM** | **chr19:17026248-17026648** |
| **20** | **36429223** | **rs1076664** | **TC** | **CC** | **CC** | **TC** | **IFF** | **chr20:36429023-36429423** |
| **21** | **25041047** | **rs2828401** | **AC** | **CC** | **AC** | **CC** | **IFF** | **chr21:25040847-25041247** |
| **22** | **26634067** | **rs9613137** | **AG** | **GG** | **GG** | **AG** | **IFF** | **chr22:26633867-26634267** |

**Suppl Table 2. Primers and probes of ddPCR**

| Primers/Probes | sequence | | |
| --- | --- | --- | --- |
| QianHT 01 F |  | AGCCAGGGAATGAAGGAA |  |
| QianHT 01 R |  | CCTCTCTCCTGCGTGAA |  |
| QianHT 01 W | HEX | ACTGAGGGCGTGGTGCTTA | BHQ1 |
| QianHT 01 M | FAM | ACTGAGGGCATGGTGCTTAG | BHQ1 |
| QianHT 02 F |  | GGGTAAGCAGGTGTCTCA |  |
| QianHT 02 R |  | GTGCCCCAGAAATCAGAA |  |
| QianHT 02 W | HEX | AGAGAAAACAAAGTGAGAGGAGGT | BHQ1 |
| QianHT 02 M | FAM | AGAGAAAACAAGGTGAGAGGAGG | BHQ1 |
| QianHT 03 F |  | GGCTACTTTGTGTTGCTCCT |  |
| QianHT 03 R |  | TCAGCAGCCATAGATCACCT |  |
| QianHT 03 W | HEX | GGCCAGATAACGGTACCAGCT | BHQ1 |
| QianHT 03 M | FAM | GGGCCAGATAATGGTACCAGCT | BHQ1 |
| QianHT 04 F |  | AGCCATCTGCCTATACTTTTA |  |
| QianHT 04 R |  | GGACCAGAGATATTGAATGACT |  |
| QianHT 04 W | HEX | CCTAAGGCTACCACCCATCATC | BHQ1 |
| QianHT 04 M | FAM | ACCTAAGGCTATCACCCATCATC | BHQ1 |
| QianHT 05 F |  | TGGGCAGACTCACTCCT |  |
| QianHT 05 R |  | GAACGCATGGTGAACAAG |  |
| QianHT 05 W | HEX | CTGCGTCTACCTTGAATTCTGAG | BHQ1 |
| QianHT 05 M | FAM | CTGCGTCTACCCTGAATTCTGAG | BHQ1 |
| QianHT 06 F |  | CTGCATGTGATAAAAGCCTAAT |  |
| QianHT 06 R |  | CCACAACTACAAGGCAACAG |  |
| QianHT 06 W | HEX | GCTCATGGGTTCTGGGGC | BHQ1 |
| QianHT 06 M | FAM | CTCATGGGCTCTGGGGC | BHQ1 |
| QianHT 07 F |  | CGCTATGTAACCCACCCT |  |
| QianHT 07 R |  | ACCCCATCACCATCCTCT |  |
| QianHT 07 W | HEX | AGGCCACACAGTTCTCTTTTAAC | BHQ1 |
| QianHT 07 M | FAM | AGGCCACACAGCTCTCTTTTAAC | BHQ1 |
| QianHT 08 F |  | AGGATGGCTGTGAAAAGAC |  |
| QianHT 08 R |  | GGTCCCAATTTCTCCACA |  |
| QianHT 08 W | HEX | GCAGTTAATAAGAAGTGTTGATGAGA | BHQ1 |
| QianHT 08 M | FAM | GCAGTTAATAAGGAGTGTTGATGAG | BHQ1 |
| QianHT 09 F |  | TATGCCTTTCCAGTTATTCAA |  |
| QianHT 09 R |  | GCCTGGCAACAAACTAATAA |  |
| QianHT 09 W | HEX | TCCTTTTGTATCCCTGGCGA | BHQ1 |
| QianHT 09 M | FAM | TCCTTTTGTGTCCCTGGCG | BHQ1 |
| QianHT 10 F |  | AATAGACCACGAGAAGGAGAC |  |
| QianHT 10 R |  | AATCTAAAGTTGCCCATCTGC |  |
| QianHT 10 W | HEX | GGTACAGCCTCGTGCCTTATT | BHQ1 |
| QianHT 10 M | FAM | GGTACAGCCTTGTGCCTTATTC | BHQ1 |
| QianHT 11 F |  | CTGCTCTTACGTCCATTCAC |  |
| QianHT 11 R |  | AGCCCATAATCCATCTCAAT |  |
| QianHT 11 W | HEX | GGGTTGGTGTGGAATTTTCTT | BHQ1 |
| QianHT 11 M | FAM | AGGGTTGGTGTAGAATTTTCTT | BHQ1 |
| QianHT 12 F |  | CCTGTGGAGTCTAAGAAATCTG |  |
| QianHT 12 R |  | GCCAGGAACAGAGAAGAGAT |  |
| QianHT 12 W | HEX | CCTTTAGTCTCAGTTGGCAGTGT | BHQ1 |
| QianHT 12 M | FAM | CCTTTAGTCTCGGTTGGCAGTG | BHQ1 |
| QianHT 13 F |  | ACCTTTTGATTCCAGGAAGA |  |
| QianHT 13 R |  | CACAGAAAGAAAGCAGATCAC |  |
| QianHT 13 W | HEX | CCCAAGCCGCCCTGAGT | BHQ1 |
| QianHT 13 M | FAM | CCCAAGCCACCCTGAGTG | BHQ1 |
| QianHT 14 F |  | AATTTTTAGTTGTGCATGTAGG |  |
| QianHT 14 R |  | CAACCATTTTCAACATTCAGA |  |
| QianHT 14 W | HEX | AAGGGGTTTGAGATGGGGTAG | BHQ1 |
| QianHT 14 M | FAM | AAGGGGTTTGCGATGGGGTA | BHQ1 |
| QianHT 15 F |  | CTTGTCCTCTACGCTGTC |  |
| QianHT 15 R |  | GGAGGTTTGGCTGATAGA |  |
| QianHT 15 W | HEX | CTTTCTGTGTTACGTTTGATCTCT | BHQ1 |
| QianHT 15 M | FAM | CTTTCTGTGTTATGTTTGATCTCTG | BHQ1 |
| QianHT 16 F |  | ACCACGCTCAGCTATAGT |  |
| QianHT 16 R |  | GGCACTTGATACACAGGA |  |
| QianHT 16 W | HEX | GACGACCCCGACACCTT | BHQ1 |
| QianHT 16 M | FAM | GGACGACCCTGACACCTT | BHQ1 |
| QianHT 17 F |  | TCACATTCCCCAACTTTAGA |  |
| QianHT 17 R |  | CAGGAAGAGGAGGTTGAAG |  |
| QianHT 17 W | HEX | ATGTCTTCTCCCTTCCTGGCC | BHQ1 |
| QianHT 17 M | FAM | ATGTCTTCTCACTTCCTGGCCT | BHQ1 |
| QianHT 18 F |  | GGGTTATCTTCCATGTCTCC |  |
| QianHT 18 R |  | GGTGCAAGGACTGAAGAAT |  |
| QianHT 18 W | HEX | GCAGGCTTGTAATGGTGGATGT | BHQ1 |
| QianHT 18 M | FAM | GCAGGCTTGTGATGGTGGATG | BHQ1 |
| QianHT 19 F |  | CACCCTGAGTAAGAGCTG |  |
| QianHT 19 R |  | GCTTCCATCCTGTGCT |  |
| QianHT 19 W | HEX | GAATGCATGAACAAATGAAGGAG | BHQ1 |
| QianHT 19 M | FAM | GAATGCATGAATAAATGAAGGAGA | BHQ1 |
| QianHT 20 F |  | GATCGAAGCTAGGAACACT |  |
| QianHT 20 R |  | GGAACAGCAAGCACAGA |  |
| QianHT 20 W | HEX | GCTCCTTGGCGTACCTCAA | BHQ1 |
| QianHT 20 M | FAM | TGCTCCTTGGTGTACCTCAA | BHQ1 |
| QianHT 21 F |  | ATTAGGATTTCATGGAGACA |  |
| QianHT 21 R |  | ACTACTTCTTCATACCTGCA |  |
| QianHT 21 W | HEX | ACATGCAAGTCTCCAAATGAC | BHQ1 |
| QianHT 21 M | FAM | ACATGCAAGTATCCAAATGACC | BHQ1 |
| QianHT 22 F |  | TGATATCCCAGGGCAGTG |  |
| QianHT 22 R |  | TGCCTGCTCTGACCAAT |  |
| QianHT 22 W | HEX | AACAGAGGTCGGGATTTGAGC | BHQ1 |
| QianHT 22 M | FAM | GAACAGAGGTCAGGATTTGAGC | BHQ1 |
| QianHT X F |  | CTTCTTGTACAATCTGGGA |  |
| QianHT X R |  | TGGTTTCACATATATCTTCTT |  |
| QianHT X W | HEX | ACTTTGGGGCTTAACGAGAA | BHQ1 |
| QianHT X M | FAM | CTTTGGGGCCTAACGAGAA | BHQ1 |
